# A high-performance 8 nV/√Hz 8-channel wearable and wireless system for real-time monitoring of bioelectrical signals

**DOI:** 10.1186/s12984-019-0629-2

**Published:** 2019-12-10

**Authors:** Konstantinos Petkos, Simos Koutsoftidis, Thomas Guiho, Patrick Degenaar, Andrew Jackson, Stephen E. Greenwald, Peter Brown, Timothy Denison, Emmanuel M. Drakakis

**Affiliations:** 1 Department of Bioengineering, Imperial College London, Prince Consort Road, London, SW7 2AZ UK; 2 Center for Neurotechnology, Imperial College London, Prince Consort Road, London, SW7 2AZ UK; 3 Institute of Neuroscience, Newcastle University, Framlington Place, Newcastle upon Tyne, NE2 4HH UK; 4 School of Electrical & Electronic Engineering, Newcastle University, Merz Court, Newcastle upon Tyne, NE1 7RU UK; 5 Blizard Institute, Barts and The London School of Medicine and Dentistry, Queen Mary University of London, 4 Newark Street, London, E1 2AT UK; 6 MRC Brain Network Dynamics Unit, University of Oxford, Mansfield Road, Oxford, OX1 3TH UK; 7 Nuffield Department of Clinical Neurosciences, University of Oxford, Level 6, West Wing, John Radcliffe Hospital, Oxford, OX3 9DU UK

**Keywords:** Neurological disorders, Bioelectrical signals, Analog front-end, High-performance, Wearable, Wireless, Bioinstrumentation

## Abstract

**Background:**

It is widely accepted by the scientific community that bioelectrical signals, which can be used for the identification of neurophysiological biomarkers indicative of a diseased or pathological state, could direct patient treatment towards more effective therapeutic strategies. However, the design and realisation of an instrument that can precisely record weak bioelectrical signals in the presence of strong interference stemming from a noisy clinical environment is one of the most difficult challenges associated with the strategy of monitoring bioelectrical signals for diagnostic purposes. Moreover, since patients often have to cope with the problem of limited mobility being connected to bulky and mains-powered instruments, there is a growing demand for small-sized, high-performance and ambulatory biopotential acquisition systems in the Intensive Care Unit (ICU) and in High-dependency wards. Finally, to the best of our knowledge, there are no commercial, small, battery-powered, wearable and wireless recording-only instruments that claim the capability of recording electrocorticographic (ECoG) signals.

**Methods:**

To address this problem, we designed and developed a low-noise (8 nV/√Hz), eight-channel, battery-powered, wearable and wireless instrument (55 × 80 mm^2^). The performance of the realised instrument was assessed by conducting both ex vivo and in vivo experiments.

**Results:**

To provide ex vivo proof-of-function, a wide variety of high-quality bioelectrical signal recordings are reported, including electroencephalographic (EEG), electromyographic (EMG), electrocardiographic (ECG), acceleration signals, and muscle fasciculations. Low-noise in vivo recordings of weak local field potentials (LFPs), which were wirelessly acquired in real time using segmented deep brain stimulation (DBS) electrodes implanted in the thalamus of a non-human primate, are also presented.

**Conclusions:**

The combination of desirable features and capabilities of this instrument, namely its small size (~one business card), its enhanced recording capabilities, its increased processing capabilities, its manufacturability (since it was designed using discrete off-the-shelf components), the wide bandwidth it offers (0.5–500 Hz) and the plurality of bioelectrical signals it can precisely record, render it a versatile and reliable tool to be utilized in a wide range of applications and environments.

## Background

**Fig. 1:**
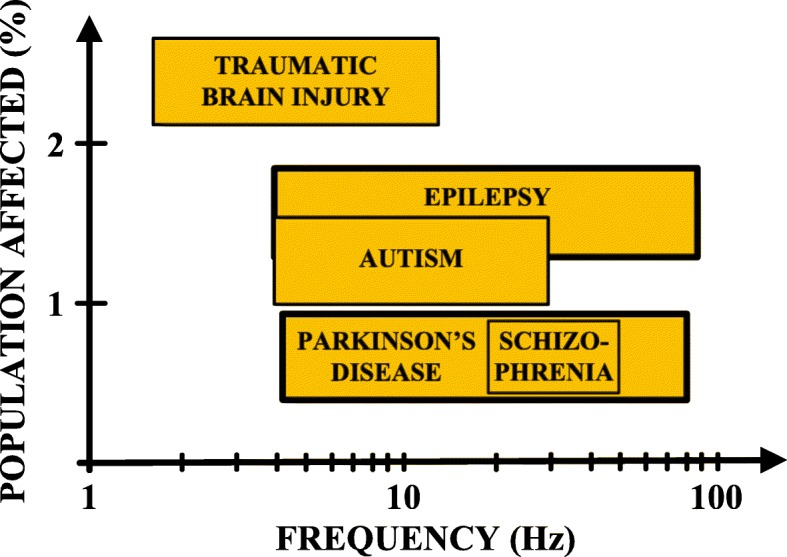
Frequency bands of abnormal phase synchrony characterizing serious neurological disorders [1, 4, 5]

Neuroelectrical activity in the brain generates oscillatory bioelectrical signals, occurring in multiple frequency bands, such as alpha (8–12 Hz), beta (13–30 Hz) and gamma (40–80 Hz) [1]. These oscillations result from the coordination or synchronization of neural activity and have been linked to a wide range of cognitive and perceptual processes [2]. However, they may also reflect abnormal function and present as key biomarkers of many serious neurological disorders, such as Parkinson’s disease (PD), epilepsy, traumatic brain injury, schizophrenia and autism [3]. Such biomarkers could improve the accuracy of diagnosis of the disease state and facilitate the correct therapy. Thus, their identification is becoming more and more crucial. Fig. 1 lists the frequency bands where abnormal phase synchrony linked to serious neurological disorders exists, and the percentage of the population affected by each disease [1, 4, 5]. Significant biomarkers that reveal the onset of severe diseases can be extracted from bioelectrical signals (Table 1).

**Table 1:** Biosignal characteristics [6]

Signal	Bandwidth	Amplitude	Invasiveness
Spikes	100 Hz–10 kHz	50–500 μV	Invasive
LFP	0.5–200 Hz	10 μV–1 mV	Invasive
EEG	0.5–100 Hz	1–20 μV	Non-invasive
ECoG	0.5–200 Hz	5–100 μV	Moderately invasive
EMG	7–500 Hz	50 μV–2 mV	Minimally or non-invasive
ECG	0.5–40 Hz (monitoring)	0.1–5 mV	Non-invasive

Recording of neurophysiological activity is an accepted medical strategy for applications ranging from seizure monitoring to neuroprosthesis [7]. Various techniques, each with a different set of tradeoffs in terms of invasiveness, spatial resolution and long-term quality and stability of chronic recording, can be used to measure neuronal activity. Single-cell recording [7–10] provides high spatial resolution, but at the expense of challenging requirements for chronic electrode–tissue interface stability, the need for preprocessing of information prior to its wireless transmission and increased amplifier power [7, 9–11]. Electroencephalography offers minimally invasive recording, but at the cost of limited spatial resolution, and the acquisition of weak signals, vulnerable to environmental noise and motion/muscle artefacts [7, 11].

In other invasive biopotential acquisition techniques, neural measurements are obtained, both on the surface of the cortex (electrocorticographic/ECoG signals) [12, 13] and from a region around an implanted electrode (LFPs). The advantage of these techniques is that they are less susceptible to chronic measurement issues and can thus offer more robust measurement of biomarkers [7, 10, 11]. They are also less susceptible to artefacts encountered in externalized surface EEG recording setups [7, 11]. Furthermore, ECoG and LFP signals encode biomarkers related to epileptic seizures [14] and the spectral decomposition of these signals can encode the necessary information for implementing an effective neuroprosthetic interface [11, 14, 15].

Increasing evidence suggests that the strength of LFP oscillations in the beta frequency band (13–30 Hz), which can be consistently picked up in the subthalamic nucleus (STN) of patients with PD, correlates with the severity of the disease and the efficacy of therapy [16, 17]. However, the last decade of LFP analysis also focused on spectral power extraction from higher frequency bands, such as high gamma (60–80 Hz) and 300 Hz (270–330 Hz) [18]. The power of these oscillations also correlates with PD motor symptoms and clinical conditions, thus being eligible as a biomarker [19].

Resonant neural response evoked by DBS, which is a rapidly expanding treatment for neurological and psychiatric diseases, is a large-amplitude neural signal that focally appears in the STN. This response is greatest in the dorsal region, which is the clinically optimal stimulation target for PD, coincides with improved clinical performance, is chronically recordable, and is present under general anesthesia [20]. These features render it as a readily utilizable electrophysiological signal and a target-specific biomarker that could potentially be used for guiding electrode implantation surgery and optimizing DBS therapy to improve patient outcomes [20]. Fasciculations, which are random muscle twitches that can be observed clinically, are of paramount importance in the diagnosis of motor neuron diseases [21]. The exact origin of these fasciculation potentials is unknown [21].

Aside from recording neuronal electrophysiological signals, acquisition of non-neuronal biological signals is also of paramount importance, especially in closed-loop neurostimulation systems [22]. Taking into account the dopaminergic-related origins of PD and biochemical basis of other neuropsychiatric diseases, the concept of integrating real-time biochemical assessments could be useful for managing dynamic fluctuations in medication effects [22]. Other non-neuronal biosignals which indicate patient movement and clinical status in real-time are peripheral physiological signals recorded using electromyographic techniques and signals recorded by non-invasive accelerometers or gyroscopes [22].

In addition, electrocardiography examines changes in cardiac electrical activity, e.g. rhythm disturbances, and is thus considered to be a crucial diagnostic modality [23, 24]. Finally, pulmonary veins (PV) play a major role in triggering atrial fibrillation (AF) in humans but the mechanisms underlying PV ectopy remain unclear [25]. Schauerte showed that it was possible to identify areas in the atria that were richly innervated with autonomic nerves, termed ganglionated plexi (GP), using high frequency stimulation (HFS) through an endovascular approach [26]. Ablation of these GPs abolished these effects [27–29]. Spontaneous PV ectopy, which is known to trigger clinical AF, may be reduced by adjunctive atrial autonomic ablation [25].

Taking into consideration that the identification of new biomarkers related to serious diseases is of paramount importance, a crucial part of a medical diagnosis system is the monitoring of bioelectrical signals. Nowadays, these signals are recorded routinely in the clinic. However, patients have to cope with the problem of limited mobility because they are connected to bulky and mains-powered instruments. This prevents the continuous monitoring of patients, restricts the signal acquisition time and deteriorates the diagnostics of serious diseases. Hence, there is a growing demand for small-sized, low-power and ambulatory biosignal recording devices [30].

The initial focus of this work was to design and assess a state-of-the-art analog front-end (AFE) that is able to: a) offer an adequate passband and dynamic range for recording a plethora of bioelectrical signals, b) successfully combine low power consumption with high performance, c) interface with both low-impedance (e.g. electrodes used in electroencephalography) and high-impedance electrodes (e.g. segmented electrodes used in DBS [31–33]), and d) provide accurate biosignal recordings that can be used to investigate the existence of possible biomarkers that characterize various diseases. The ultimate aim was to use the afore-described versatile and high-performance AFE as the fundamental building block for producing a wearable and wireless device that could be used by clinicians, either independently or functionally integrated with existing medical biosignal recording systems, to track a patient’s clinical state.

## Methods

### Design requirements and implementation of the AFE

In order to achieve the goals specified in the introduction, a number of strict specifications for signal acquisition were imposed. These specifications are summarized in Table 2. Clearly, a high gain and common mode rejection ratio (CMRR) along with low noise levels are required in order to ensure precise recordings of weak bioelectrical signals. Moreover, a relatively wide dynamic range in combination with adequate dc offset rejection capabilities are required in order to prevent the output of the instrument’s AFE from saturation caused by the dc offsets stemming from the recording electrodes.

**Table 2:** Summary of key performance specifications related to the AFE of the proposed instrument

Property	Value	Units/Comments
Gain	≥ 40	dB
Integrated noise	≤ 300	nVrms (0.5 to 500 Hz)
CMRR	≥ 100	dB
DC tolerance	≥ 50	mV
Current consumption	≤ 5	mA
Input dynamic range	≥ ± 2	mV
Input impedance	≥ 1	GΩ
High-pass knee frequency	0.5	Hz
Low-pass knee frequency	500	Hz

**Fig. 2:**
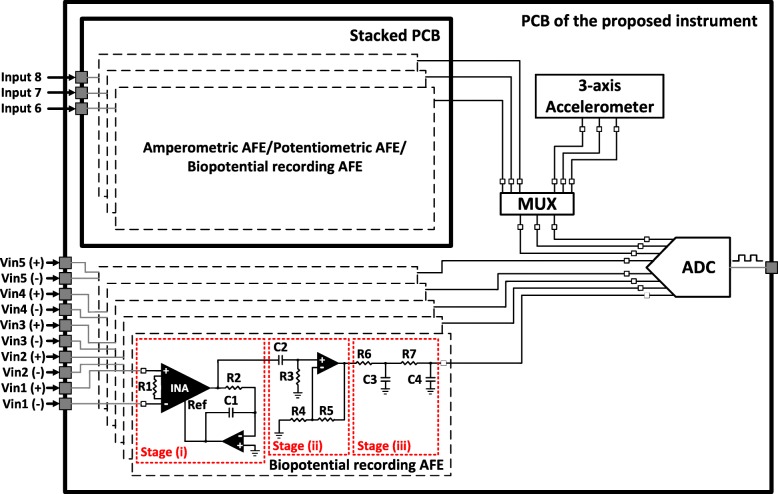
Block diagram showing the overall AFE and the ADC section of the proposed instrument. The overall AFE consists of eight channels (five channels on the main printed circuit board (PCB) of the instrument and another three channels on a stacked PCB that is adjusted on two headers located on the main PCB). The biopotential recording AFE consists of three stages: (i) a differential pre-amplification stage with high-pass characteristics, (ii) an active, 1st order high-pass filter that enhances the dc offset rejection capabilities of the AFE and (iii) a passive, 2nd order low-pass filter that defines the passband of the AFE and also serves as an anti-alias filter for the ADC stage, which follows in the signal chain. The inputs of the first five channels are differential, whereas the inputs of channels 6, 7 and 8 can either be differential or single-ended depending on the type of biosignals (either amperometric or potentiometric or biopotential signals or a combination of them) the stacked PCB is intended to record

To meet the requirements for data acquisition, we designed and implemented a biopotential recording AFE architecture consisting of three main stages (Fig. 2): (i) a differential pre-amplification stage with high-pass characteristics; (ii) an active, 1st order high-pass filter that enhances the dc offset rejection capabilities of the AFE; and (iii) a passive, 2nd order low-pass filter that defines the passband of the AFE and also serves as an anti-alias filter for the analog-to-digital converter (ADC) stage, which follows in the signal chain.

The pre-amplification stage consists of a low-noise instrumentation amplifier (INA) (model AD8422, Analog Devices, USA). Taking into consideration that neural signals, such as EEG signals and LFPs, are characterized by very low amplitudes (typical range: 1–50 μV [16]), a high CMRR in the front-end amplifier is required in order to increase the signal-to-noise ratio (SNR) of the recording instrument.

Next, to eliminate dc offsets originating from the electrode-electrolyte/tissue interface [34], an active feedback integrator [35], which functions as a 1st order high-pass filter with a knee frequency of 0.5 Hz, was introduced in the front-end of the device. Crucially, this engineering design decision along with the choice of introducing a high gain (= 40 dB) on the front-end INA chip, allows us to fully exploit the high CMRR offered by the AD8422 INA chip (134 dB for a gain of 40 dB). Moreover, since there is no passive filtering network before the front-end amplifier and the gain of the first stage is sufficiently high (equal to 40 dB) - thus allowing the effective noise factor to be the noise factor of the first stage without an impact on the subsequent stages [36, 37] - the input-referred noise of the designed AFE is expected to approximate the measured input-referred noise reported in the datasheet of the AD8422 chip (≈ 8 nV/√Hz).

The second stage (see Fig. 2) includes an active 1st order high-pass filter, which provides an amplification of 20 dB. The role of this filter is to enhance the dc offset rejection capabilities of the overall AFE and offer the additional gain required to better exploit the full scale voltage range of the ADC (± 2.5 V). Finally, the strict area and power consumption requirements imposed on the AFE design led to the introduction of a passive 2nd order low-pass filter as the third stage of the AFE, because it can be implemented with fewer components in comparison with an active low-pass filter of the same order.

Referring to Fig. 2, resistor R_1_ determines the gain of the front-end INA, while resistor R_2_ and capacitor C_1_ determine the cut-off frequency of the 1st order high-pass filter introduced in the first stage of the AFE. Next, resistor R_3_ and capacitor C_2_ define the cut-off frequency, while the ratio of resistors R_4_ and R_5_ define the gain (1 + R_5_/R_4_) of the 1st order high-pass filter located in the second stage of the AFE. Finally, resistors R_6_, R_7_ and capacitors C_3_, C_4_ determine the cut-off frequency of the 2nd order low-pass filter existing in the third (final) stage of the AFE architecture.

The first stage (differential pre-amplification) is supplied with ±5 V to ensure that an adequate headroom is provided and eliminate the risk of saturation coming from electrode dc offsets. However, the second stage (active high-pass filter) is supplied with ±2.5 V to be able to interface with the high-performance and low-power commercial ADC chip (model ADS1298, Texas Instruments, USA) that follows in the signal chain. The fundamental building block for the design of the filtering stages is the operational amplifier ADA4522 by Analog Devices. Finally, it should be noted that the resistors and capacitors included in this three-stage AFE architecture are characterized by a tolerance of 0.1 and 10% respectively.

As shown in Fig. 2, five recording channels of the proposed instrument have been designed according to the above-described AFE architecture (entitled “biopotential recording AFE” in Fig. 2). These five channels culminate in five out of eight channels of the ADC chip and can record a wide variety of bioelectrical signals, such as EEG, EMG, ECG, ECoG, LFP signals, PV ectopic activity and evoked resonant neural activity (ERNA). Furthermore, the proposed instrument has been designed to record some additional biosignals, such as acceleration signals, which are recorded by an analog three-axis accelerometer (ADXL335, Analog Devices, USA) located on the instrument’s main PCB, or signals stemming from amperometric and potentiometric biosensors, which can be recorded by three auxiliary AFEs located on a stacked PCB that is adjusted on the main PCB of the instrument.

In other words, many types of stacked PCBs could be designed, each one of them providing the ability to record different types of bioelectrical signals (amperometric/potentiometric/biopotential). The nature of the biosignals of interest would determine the type of the stacked PCB to be placed on top of the main PCB. The user can determine through software the type of biosignals (either acceleration signals or analog signals stemming from the three auxiliary AFEs located on the stacked PCB) to be digitized by the last three channels of the ADC chip. At this point, it is important to clarify that the addition of three low-power auxiliary AFEs (on the stacked PCB) offers flexibility on the type of biosignals that can be recorded by the last three channels of the ADC chip without significantly affecting the power consumption of the overall system.

Finally, the above-described low-power three-axis accelerometer, which is mainly intended to be used in applications where tremor activity of patients with PD or essential tremor needs to be monitored, is characterized by an output sensitivity of 250 mV/g and a noise of approximately 840 μgRMS (X, Y axis) and 1.7 mgRMS (Z axis) for an available bandwidth of 10 Hz. The reason behind the selection of a limited bandwidth (= 10 Hz) for the acceleration measurements lies in the fact that: a) tremor frequency of patients with PD ranges from 3 to 8 Hz [38] and b) the lower the available bandwidth the better the resolution of the accelerometer.

### Architecture of the overall system

**Fig. 3:**
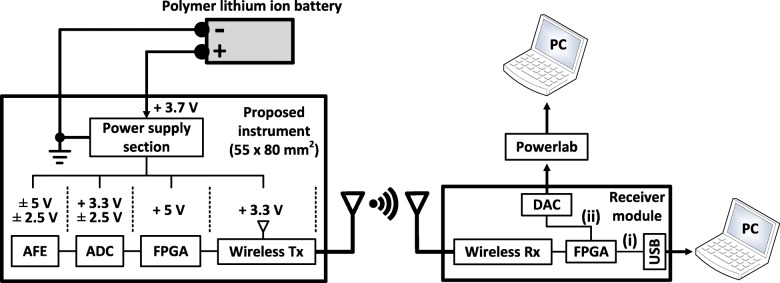
Block diagram showing the overall architecture of the proposed instrument. The data received by the receiver module can either be directed i) to a personal computer (PC) through universal serial bus (USB) 2.0 interface, or ii) to a commercially available data acquisition system (e.g. Powerlab) that digitizes and depicts the data on a PC. Path (ii) ensures that the proposed instrument can functionally integrate with existing medical biosignal acquisition systems, if required

As shown in Fig. 3, the overall system consists of an 8-channel AFE – illustrated in Fig. 2 - followed by the 8-channel, simultaneous sampling, 24-bit, delta-sigma ADS1298 chip, which further amplifies (the gain settings of the built-in programmable gain amplifier are 1, 2, 3, 4, 6, 8, and 12 V/V), if required, and digitizes the analog outputs of the biosignal recording channels. Next, a field-programmable gate array (FPGA) module (Spartan 3e – 48 MHz) controls the communication between the ADS1298 chip and the radio transceiver, which is the final stage of the system design.

Regarding wireless transmission, the communication protocol that was selected for the wireless transmission of the recorded data is the IEEE 802.15.4 protocol (the AT86RF231 radio transceiver chip from Microchip Technology was used) because: 1) similarly to Bluetooth (IEEE 802.15.1), it is suitable for low data rate applications with limited battery power (such as portable, battery-operated devices like the one presented in this work), due to its low power consumption [39], and 2) it offers extremely good bit error rate (BER) performance at low SNRs [40] (the BER performance of the 802.15.4 transmission is between 7 and 18 dB better than that of Bluetooth [40]), which is crucial since several radio services exist in a typical clinical setting, where the proposed instrument is intended to be used. Moreover, the λ/4 monopole, 2.4 GHz antenna, which is characterized by a length of 21 mm, a nominal gain of 0 dBi, an omni-directional design and sub-miniature version A (SMA)-plug fixing, was chosen since it combines small size, low cost and high performance.

**Fig. 4:**
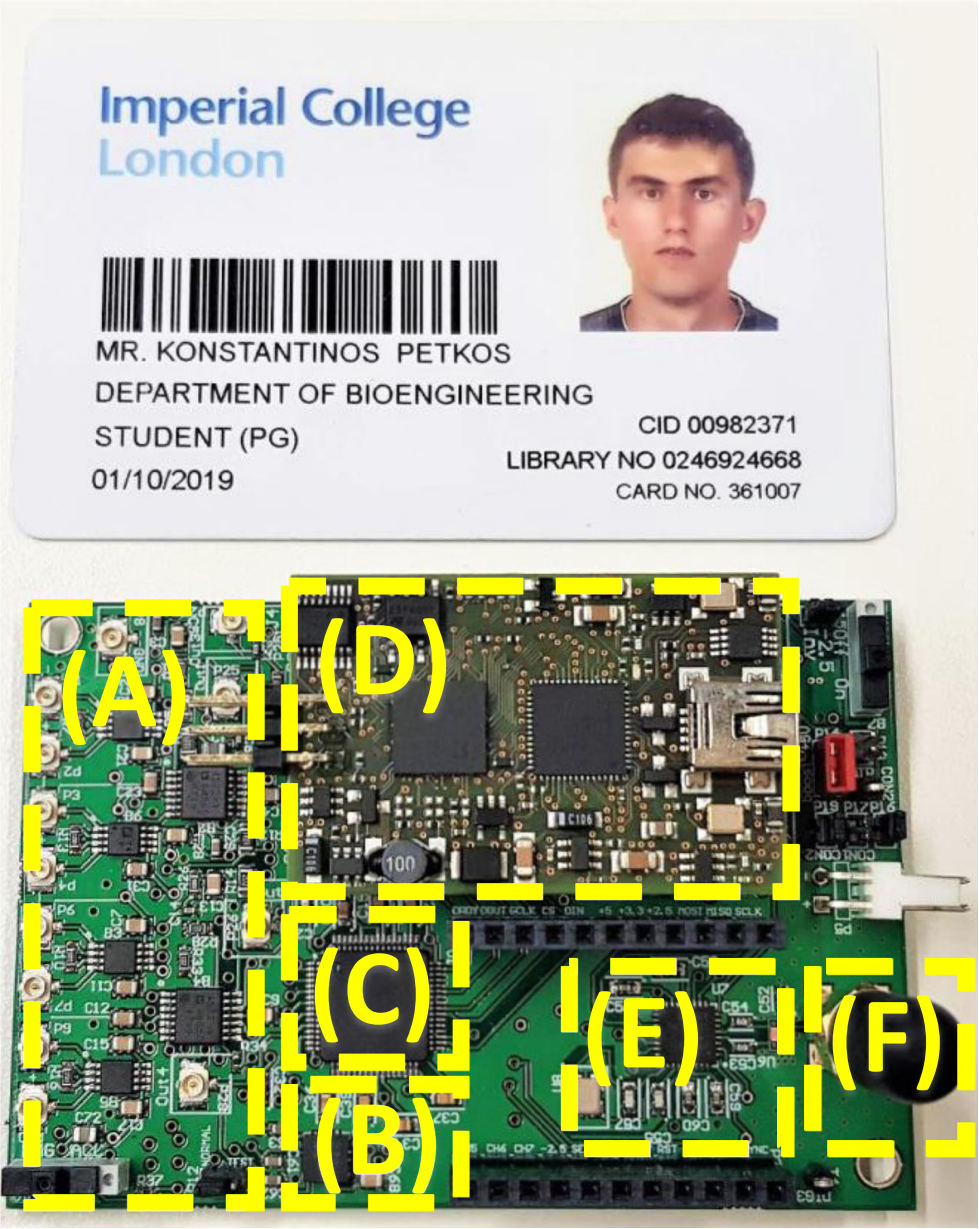
Structure of the proposed instrument. The instrument’s architecture includes: **(a)** a high-performance, eight-channel (five channels on the main board and another three channels on a stacked PCB that is adjusted on the two headers shown in the picture) AFE, **(b)** an analog, low-power 3-axis accelerometer, **(c)** an eight-channel, 24-bit ADC, **(d)** an FPGA module, **(e)** a 2 MBps Zigbee transceiver, and **(f)** a 2.4 GHz antenna

The power supply section of the instrument includes a high-efficiency, step-up DC-DC switching regulator (LM2623, Texas Instruments, USA), which is suitable for battery-powered and low input voltage systems. This regulator converts the + 3.7 V supplied by the battery to + 5 V, which is required for the operation of the AFE and the FPGA. Furthermore, the voltage regulator chip TPS7A8001 (Texas Instruments, USA), the low-noise regulated switched-capacitor voltage inverter LM27761 (Texas Instruments, USA) and the voltage converter LM2662 (Texas Instruments, USA) provide all the voltage levels (± 2.5 V, + 3.3 V and - 5 V) required for proper operation of the remaining parts of the device. The proposed instrument is powered by a 1 Ah lithium battery and can provide more than eight hours of continuous wireless biosignal transmission. Finally, its size approximates the size of a business card (55 × 80 mm^2^), as shown in Fig. 4.

By carrying out the circuit design using discrete components we reduce the barrier to manufacturing and replication. Since these components are recommended by their manufacturers for being used in medical instrumentation, their incorporation in the designs of medical devices that are intended to be used in the clinic: 1) maximizes the possibilities of successfully completing the relevant clinical tests, and 2) enables a faster acquisition of the appropriate medical approvals, and thus a faster launch into the market.

Regarding the research outcomes of our work, by using (very high-performance) discrete components in our design instead of designing an application specific integrated circuit (ASIC), our aim is to provide a low-noise instrument (with a noise performance that is better than the noise performance provided by existing devices, such as ASICs existing in the literature, commercial devices and academic works based on discrete off-the-shelf components) that can achieve a more complete sampling of the physiomarker space. Next, based on what is discovered, we can define a bespoke ASIC that would provide the resolution required within the power constraints of a miniaturized wireless device (either implantable or just wearable).

The investigational character of our device also led us to maintain the provided number of channels at a relatively moderate level (=8). Since the wireless protocol we use can support much larger data payloads than the one we produce and transmit with the 8-channel architecture, the best approach for increasing the channel count of our device (if required) is to redesign the proposed instrument with more channels (≥16) and maintain the single transmitter-single receiver system architecture. In this way, challenges in the real-time character of the system or data synchronization issues that may emerge in architectures where multiple transmitters (with one or more receivers) exist can be avoided.

Regarding the architecture of the receiver module (see Fig. 3), it consists of: a) the AT86RF231 chip that receives the data transmitted by the proposed instrument, b) an FPGA that can direct the data either to a PC through the USB 2.0 interface that is provided by the FPGA module (path (i) in Fig. 3), or to a commercially available 16-bit digital-to-analog converter (DAC) chip (AD5362, Analog Devices, USA - path (ii) in Fig. 3). The analog output of the DAC chip can then be digitized and depicted on the PC by a commercial data acquisition system (e.g. Powerlab 16/35 that is used in this study). The second path (FPGA-DAC-Powerlab) was added in the system architecture to ensure that our instrument can functionally integrate with existing commercial devices (e.g. Powerlab 16/35) and be used in medical studies.

### Design considerations for low-noise biosignal recordings in a noisy clinical setting

As far as the noise sources at low frequencies are concerned, it is well-accepted that the most important source of electromagnetic interference (EMI) at low frequencies is the 50 Hz noise from the mains. In order to protect our system from the 50 Hz interference, we implemented, cumulatively, the following strategy: a) the device was always battery-powered during the experiments to prevent the recorded biosignals from being contaminated by the strong 50 Hz interference (and its harmonics) stemming from the mains, b) an INA with a high CMRR value (> 130 dB) and a high input impedance (200 GΩ, 2 pF) was chosen to be the first stage of the designed biopotential recording AFE. These two features of the INA (high CMRR and input impedance values) suppress interference originating from the mains, c) the front-end INA further attenuates the 50 Hz noise due to the inherent cancellation of even-order harmonics it offers (since it’s a differential system).

As far as the noise sources at high frequencies are concerned, in order to protect our system from high-frequency EMI (e.g. radio frequencies, operating frequencies of medical devices used in the clinic, such as ultrasound, MRI devices etc) existing in the clinical setting, we implemented, cumulatively, the following strategy: a) a passive, 2nd order low-pass filter at 500 Hz was placed in the AFE of our instrument. This filter significantly attenuates high-frequency (>tens of kHz) noise components, b) a passive, 1st order EMI (low-pass) filter at 3 MHz is included in the ADS1298 chip, which is the ADC of our system. This filter further attenuates high-frequency (>tens of MHz) noise components, c) a high-performance INA with high CMRR values was placed at the first stage of the designed AFE. As a result, high-frequency noise components (present in the ambient environment) that appear as common-mode signals at both inputs of this front-end INA are adequately suppressed by its high CMRR (> 80 dB at 100 kHz), and d) during the biosignal (ExG) acquisition experiments, the cables that were used to connect the ExG electrodes to our recording device were twisted (whenever practically possible) and wrapped in foil to minimize the effects of noise on the recorded bioelectrical signals.

It should be clarified here that during all the experiments (both ex vivo and in vivo) presented in this paper, the PCB of our recording device was not placed in any enclosure that could function as a Faraday cage (it was thus completely exposed to external noise/EMI) in order to assess the worst case scenario. Of course, in a real clinical setting a proper plastic enclosure with EMI/RFI copper conductive coating (that functions as a Faraday cage) has to be used to host the designed PCB and protect the electronics from external noise/interference sources (coating manufacturers often specify an attenuation of more than 75 dB from 1 MHz to 1 GHz).

## Results

In this section, a number of strict tests, which are performed on the biopotential recording AFE to assess its performance in terms of noise, linearity and temporal response, are presented and analyzed. Furthermore, indicative ex vivo recordings of EEG, ECG, EMG, acceleration signals and fasciculations that were acquired from two healthy subjects using the proposed instrument, are reported. Moreover, in vivo recordings of weak LFPs, which were wirelessly acquired in real time using DBS electrodes implanted in the thalamus of a non-human primate, are also presented. Finally, raw anonymized data including extremely weak LFP signals, ERNA and PV ectopic activity, which were previously recorded by approved wired instruments during invasive experimental sessions are used to evaluate the performance of the proposed instrument against high-performance commercial biopotential acquisition systems. In this study, the above-described raw anonymized data are presented to the input of the instruments that are examined in each series of experiments by means of a commercial waveform generator (Agilent 33220A).

The results shown in this section were derived from signal recordings that took place in a university laboratory, which is vulnerable to noise/EMI originating from a wide variety of devices that are in operation (e.g. servers, ultrasonic cleaners, air fume hoods, laboratory pumps for fluid or gas transfer, a variety of automatic heating/cooling elements and systems, several other electric appliances such as fridges etc). Similar comments hold for the in vivo experiments reported here which took place at Newcastle University in a high-tech space which can be characterized as an ICU for non-human primates that contains a plethora of sized monitoring devices.

Additional experiments conducted in an active operating theatre (Hammersmith Hospital, London, UK), which is primarily used for cardiac interventions and procedures, show that the instrument’s input-referred noise levels remain unaffected when the instrument is placed (again without making use of any enclosure that could function as a Faraday cage) in a noisy clinical environment. Hence, it is clear that the high CMRR of our AFE in conjunction with the analog (low-pass) filtering strategy followed in the AFE design of our device successfully suppress external EMI and thus prevent noise from being coupled into the physiological measurements. Finally, no 50 Hz interference was present in the amplitude spectrum of the acquired noise recordings.

### Transfer function and input referred noise

**Fig. 5:**
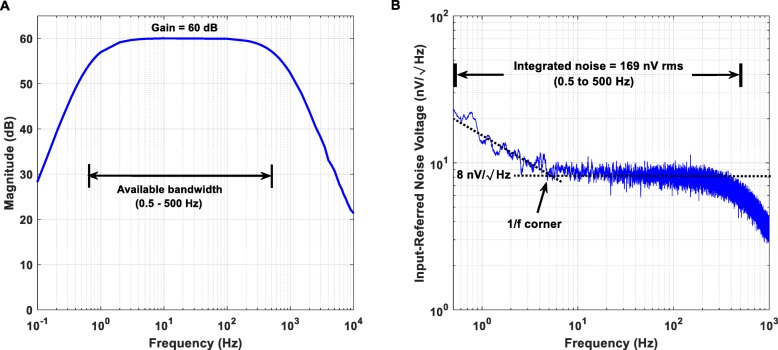
Measured Bode magnitude plot **(a)** and input-referred noise **(b)** of the proposed instrument’s biopotential recording AFE. **(a)** The biopotential recording AFE provides a passband between 0.5 and 500 Hz. The roll-off of the analog high- and low-pass filters equals + 10 dB/Oct and − 40 dB/decade, respectively. **(b)** Noise power spectral density estimate in the passband for the biopotential recording AFE is 8 nV/√퐻푧Hz, with the residual 1/f corner estimated at roughly 5 Hz

From the measured Bode magnitude plot shown in Fig. 5a, it is clear that the proposed instrument’s AFE provides a passband between 0.5 and 500 Hz and achieves the desired gain of 60 dB. The roll-off of the high- and the low-pass filters equals + 10 dB/Oct and − 40 dB/decade, respectively.

Since there is no passive filtering network before the front-end AD8422 INA chip and the gain of the first stage is sufficiently high (equal to 40 dB), the input-referred noise of the designed AFE should approximate the measured input-referred noise reported in the datasheet of the AD8422 chip. The integrated noise of the proposed instrument’s AFE over the frequency range 0.5–500 Hz was measured and found to be equal to 169 nV rms. According to Fig. 5b, the noise power spectral density estimate in the passband for the designed AFE is 8 nV/√*Hz*, with the residual 1/f corner estimated roughly at 5 Hz. Indeed, these measured results are in agreement with the noise measurements reported in the datasheet of the front-end AD8422 INA chip.

### Total harmonic and intermodulation distortion

**Fig. 6:**
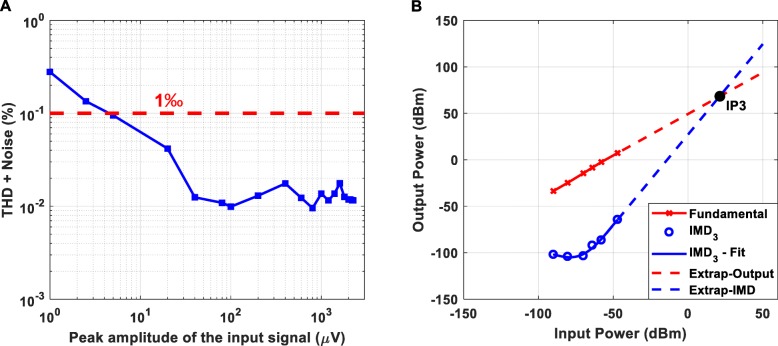
Total harmonic distortion + Noise **(a)** and third order intermodulation distortion (IMD_3_) **(b)** of the proposed instrument’s AFE (gain = 60 dB). **(a)** Taking into consideration that the available dynamic range of the AFE is from 1 μV peak to 2.3 mV peak, it is clear that the achieved THD + Noise is less than 0.3%. **(b)** The two tones applied to the AFE of the proposed instrument were f_1_ = 4.9 Hz and f_2_ = 5.1 Hz. The output power of a single fundamental tone (in dBm - red line in the graph) and the relative power of the IMD_3_ products referenced to a single tone (blue circles) are plotted against the applied input power. The third-order intercept line (dashed blue line) is extended to intersect the extension of the fundamental output signal line (dashed red line). This intersection is termed the third order intercept point IP_3_. The calculated IP_3_ exhibits a relatively high value, which is desired, since the higher the IP_3_ value the better the linearity of the amplifier and the weaker the output intermodulation products that will be generated at the amplifier’s output

The measured total harmonic distortion (THD) plus noise of the proposed instrument’s AFE (gain = 60 dB) is presented in Fig. 6a. Taking into consideration that the available dynamic range of the AFE is from 1 μV peak to 2.3 mV peak, it is clear that the achieved THD plus noise is less than 0.3%. As shown in Fig. 6b, the third order intercept point (IP_3_) of the proposed instrument’s AFE is characterized by a desirable relatively high value (the higher the IP_3_ values the more linear the amplifier and the weaker the distortion products at its output).

### Step and impulse response

The step response of a filter, which is the integral of the impulse response, is useful in determining the envelope distortion of a modulated signal [41]. The two most important characteristics of a filter’s step response are the overshoot and the ringing. Overshoot must be minimal for good pulse response and ringing must decay as fast as possible, so that interference with subsequent pulses is avoided. Transient response curves cannot provide a completely accurate estimation of the output since, in practice, signals typically are not made up of impulse pulses or steps. However, these curves constitute a convenient figure of merit so that transient responses of various filter types can be compared on an equal footing [41].

**Fig. 7:**
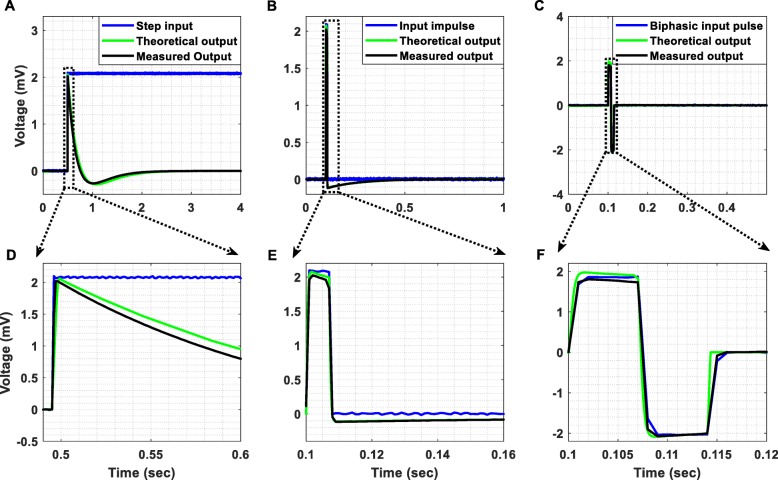
(**a**) Step response, (**b**) impulse response and (**c**) response to a biphasic pulse, which characterize the proposed instrument’s AFE. (**d**) The step response of the AFE, which initially exhibits an undershoot of 250 μV, is free of ringing (there is no decaying oscillatory activity after the undershoot). (**e**) The impulse response of the AFE exhibits a relatively fast settling. (**f**) The response of the AFE to a biphasic pulse exhibits a significantly faster settling in comparison to its corresponding impulse response (shown in (**e**)). In all cases, the output voltage (black line) is presented after removing the gain of 60 dB that is applied by the biopotential recording AFE

The step response of our AFE (Fig. 7a), which initially exhibits an undershoot of 250 μV, is free of ringing and the impulse response (Fig. 7b) exhibits a relatively fast settling. Another important test for evaluating the temporal response of the designed AFE is to inject a biphasic pulse to its input. As anticipated, the response of our AFE to a biphasic pulse (see Fig. 7c) exhibits a significantly faster settling in comparison to its impulse response. In all cases, the theoretical responses (based on the transfer function of the overall system – plotted in solid green line in Fig. 7) approximate the measured ones (plotted in solid black line in Fig. 7). Finally, it is important to note here that the minimum slew rate achieved by the front-end amplifiers of the proposed instrument is 0.8 V/μs.

### Ex vivo recordings of ExG (EEG, EMG and ECG) signals and fasciculations

In order to assess the low-noise recording capabilities of the designed instrument, we recorded physiological signals that do not require invasive measurement techniques and measured the achieved SNRs. Another objective of these biosignal recordings was to show that in spite of the high gain (60 dB minimum – 40 dB from the front-end INA and another 20 dB from the active high-pass filter existing at the 2nd stage of the biopotential recording AFE, see Fig. 2) and the relatively small dynamic range (± 2.3 mV) that characterize the biopotential recording AFE, it is capable of rejecting the dc offsets coming from the electrodes and thus avoiding saturation.

**Fig. 8:**
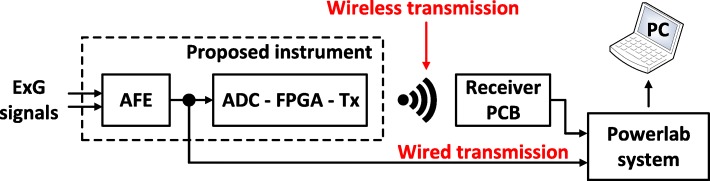
Measurement setup for comparing the quality of biosignals recorded using two different methods. In the first method (wired transmission), the signals are recorded by the AFE of the proposed instrument and are directly digitized and depicted on the computer by a commercial instrument (Powerlab 16/35). In the second method (wireless transmission), the signals are recorded by the proposed instrument, are wirelessly transmitted to a receiver module and are depicted on the computer by the Powerlab 16/35 hardware

The measurement setup (Fig. 8) used in this series of experiments allows for the comparison of the quality of signals recorded using two different methods. In the first method (wired transmission), the signals are recorded by the AFE of the proposed instrument and are directly digitized and depicted on the computer by a commercial instrument (Powerlab 16/35) bypassing all the stages of the proposed instrument located after its AFE (Fig. 8). In the second method (wireless transmission), the signals are recorded and digitized by the proposed instrument, are wirelessly transmitted to the receiver module and are depicted on the computer by the Powerlab 16/35 hardware. This setup aims at confirming that the proposed instrument can functionally integrate with existing commercial devices used in clinical studies and provide faithful wireless transception. It is clear that this setup can be perceived as the worst case scenario, since, in the wireless communication method, the recorded biosignals have to be digitized by the proposed instrument, converted back to analog from the DAC of the receiver module and then digitized again by the ADC of the Powerlab hardware.

One of the aims of these experiments was to record alpha wave (7.5–12.5 Hz) activity, which is accepted as the most prominent proof of an instrument’s capability to measure EEG signals. The setup included three electrodes, which were positioned as follows: 1. F3 – Ground Electrode, 2. F4 – Reference Electrode, and 3. O2 – Recording Electrode. In this test, an increased alpha wave activity is expected to appear in the spectrum of the EEG signals recorded when the subject’s eyes are closed.

In principle, EMG signals are recorded using either minimally invasive or skin surface electrodes. For this study, three skin surface disposable solid gel electrodes (contact size 15 × 20 mm), produced by Unimed, were placed at the following upper limb positions:

1. Palmaris longus muscle – Recording Electrode.
2. Metacarpal bones – Reference Electrode.
3. Proximal phalanx – Earth Electrode.

Regarding ECG signal acquisition, a simple three electrode monitoring setup was prepared by using two electrodes for active monitoring and a third one as ground electrode [42]. The electrodes were used in lead I (RA-LA) configuration leading to a bipolar signal acquisition. The ground electrode was placed on the right leg ankle. The electrodes used for the signal acquisition are the Max-TAB resting electrodes (contact size 20 × 24 mm), produced by Unimed.

**Fig. 9:**
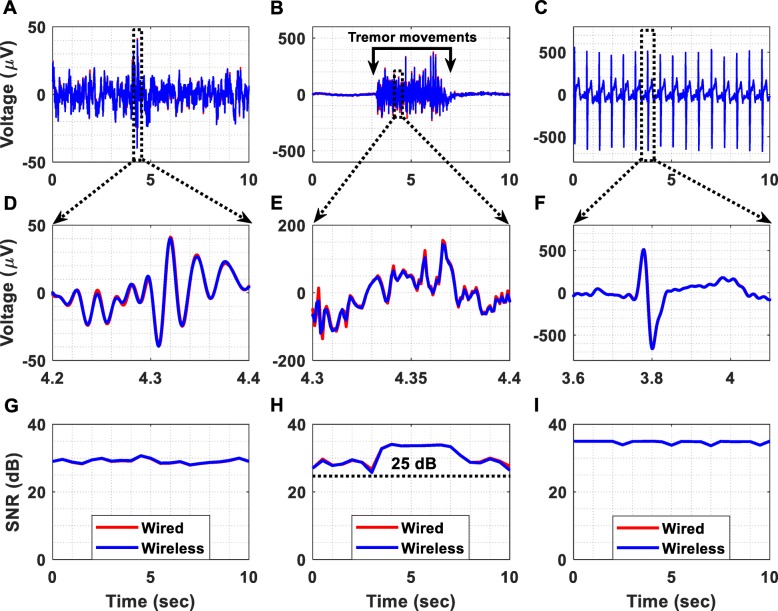
Biosignal acquisition using the setup presented in Fig. 8. The applied gain was 60 dB and the sampling frequency was equal to 1 kSPS. **(a)** Wired vs wireless EEG acquisition. **(b)** Wired vs wireless EMG acquisition from the palmaris longus muscle. **(c)** Wired vs wireless ECG acquisition. **(d)** Detailed view of the wireless and wired time-domain EEG recordings. **(e)** Detailed view of the wireless and wired time-domain EMG recordings. **(f)** Detailed view of the wireless and wired time-domain ECG recordings. **(g)** The SNR of the EEG signals was measured and found to be continuously higher than 25 dB. **(h)** The SNR of the EMG signals was measured and found to be continuously higher than 25 dB. **(i)** The SNR of the ECG signals was measured and found to be continuously higher than 30 dB

The ExG (EEG, EMG and ECG) signals, which are exhibited in Fig. 9 after removing the applied gain of 60 dB, were recorded at 1 kSPS. Figure 9a illustrates a time-domain EEG recording acquired using both of the previously described methods (wired and wireless). Regarding the EMG measurement (Fig. 9b), the high amplitude signal was recorded while the subject was producing tremor movements. Moreover, Fig. 9c shows that ECG signals were successfully recorded. In order to examine the performance cost introduced by the wireless transmission method, the normalized root mean square error (RMSE) between the time-domain recordings acquired using the two methods (wired and wireless transmission methods) was calculated and found to be equal to 1.7, 1.1 and 0.39% for the EEG, EMG and ECG measurement setups, respectively. These errors can be considered tolerable taking into account that this experiment assesses the above-described worst-case scenario.

**Fig. 10:**
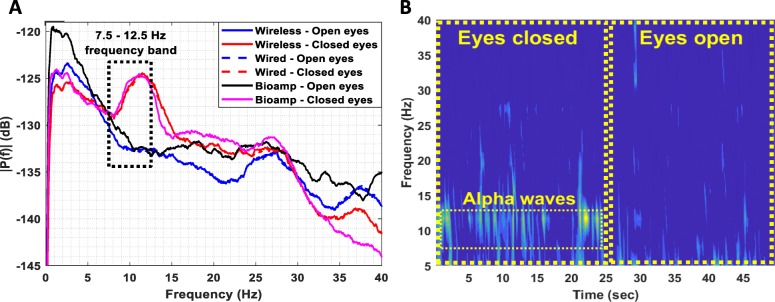
(**a**) Amplitude spectrum (the reference level of amplitudes equals 1 V) of EEG signals recorded by the proposed instrument when the subject’s eyes are open and when they are closed. A high-performance commercial bioamplifier (Powerlab 26 T, ADInstruments) was also used as a reference instrument in this series of experiments. It is clear that in the 7.5–12.5 Hz band the amplitude spectrum of the EEG signals recorded when eyes are closed is significantly higher than the amplitude spectrum of the signals recorded when eyes are open. Furthermore, the results acquired using the wireless transmission method: 1) are in full agreement with the results acquired using the wired transmission method, and 2) are in full agreement with the results acquired using the commercial bioamplifier. (**b**) EEG spectrogram calculated from the EEG data wirelessly recorded by the proposed instrument. Alpha waves in the 7.5–12.5 Hz band during the eyes closed period are clearly visible

Figure 9g, h and i illustrates the achieved SNR over time during the EEG, EMG and ECG recording sessions, respectively. It is clear that, during all recording sessions, the achieved SNR values were higher than 25 dB. The alpha waves test, presented in Fig. 10, shows that in the band 7.5–12.5 Hz, the amplitude spectrum of the EEG signals recorded when the eyes are closed is significantly higher than the amplitude spectrum of the signals recorded when the eyes are open (Fig. 10a). The same conclusion is derived from Fig. 10b where alpha waves in the band 7.5–12.5 Hz, during the eyes closed period, are clearly visible.

Another important observation is that the amplitude spectrums of the EEG signals recorded using the wired and wireless data transmission methods, not only approximate each other, but also they are in perfect agreement with the amplitude spectrum recorded (at 1 kSPS) by a high-performance commercial bioamplifier (Powerlab 26 T, ADInstruments) (see Fig. 10a). This finding suggests that the proposed instrument can provide reliable recordings even when its enhanced recording capabilities are not fully exploited. Indeed, in both methods adopted in this series of experiments (wired and wireless), the biosignals recorded by the proposed instrument were finally digitized by the Powerlab 16/35 system, which provides 16-bit resolution for the analog-to-digital conversion process (whereas our instrument can provide 24-bit resolution, if used independently).

**Fig. 11:**
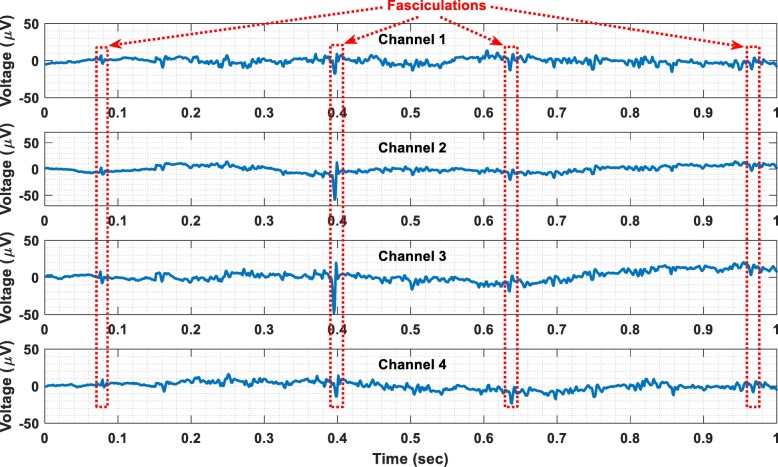
Wireless recording of benign fasciculations

Finally, benign fasciculations have been recorded from a healthy subject using the wireless transmission method. The recorded fasciculations are shown in Fig. 11. The electrodes that were used for this recording session are the same with the ones used to record EMG signals. Referring to Fig. 11, it is clear that the proposed instrument can successfully record both weak and strong fasciculations. Hence, it could be used as a research tool to distinguish benign fasciculations and those related to amyotrophic lateral sclerosis (ALS) on the basis of their waveforms or firing characteristics [21]. It could also be used as a wireless, home monitoring device for increasing the biosignal acquisition time and thus enhancing the diagnostics of ALS.

### In vivo recordings of LFPs

**Fig. 12:**
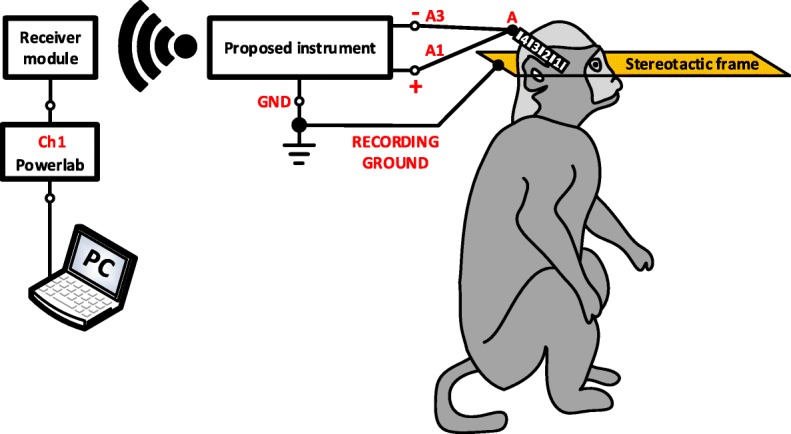
Experimental setup for evaluating the recording capabilities of the proposed instrument in vivo. A DBS electrode (electrode A, model DB-2201, Boston Scientific Neuromodulation) was implanted into the thalamus of an anaesthetised non-human primate. LFP signals were differentially recorded through contacts 1 and 3 of electrode A. The non-human primate was under anaesthesia with the head held in a primate stereotactic frame, which was connected to the ground of the recording system. The LFP signals were digitized by our instrument at a sampling frequency of 1 kSPS and were wirelessly transmitted to the receiver module (wireless transmission method – described in Fig. 8)

**Fig. 13:**
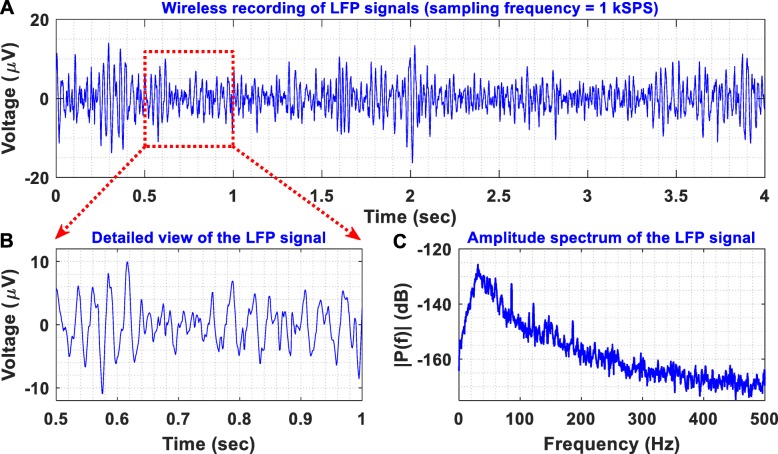
(**a**) Differential LFP recordings acquired from the thalamus of an anaesthetised non-human primate with the experimental setup illustrated in Fig. 12. (**b**) A detailed view of the recorded LFPs reveals their small amplitudes (< 20 μV peak). (**c**) Amplitude spectrum of the recorded LFPs. Clearly, the proposed instrument can wirelessly provide low-noise recordings of weak LFP signals in a noisy clinical environment

To provide an in vivo proof-of-function, we recorded LFPs from the thalamus of a non-human primate, at the end of a non-recovery procedure that was performed for the primary purpose of another ongoing study. A female rhesus macaque was anesthetised with a ketamine/midazolam/alfentanil infusion and a segmented DBS electrode (electrode A, model DB-2201, Boston Scientific Neuromodulation) was implanted into the thalamus as shown in Fig. 12. LFP signals were differentially recorded through contacts 1 and 3 of electrode A (illustrated as A_1_ and A_3_ in Fig. 12, respectively). The non-human primate was under anaesthesia during the entire experiment with the head held in a primate stereotactic frame, which was connected to the ground of the recording system. The LFP signals recorded by our instrument were digitized at a sampling frequency of 1 kSPS and were wirelessly transmitted to the receiver module. Then, they were depicted on a computer by the Powerlab data acquisition system (wireless transmission method - described in Fig. 8). As shown in Fig. 13, the proposed instrument can wirelessly provide low-noise recordings of weak LFP signals in a noisy animal clinic environment.

### Recording of resonant neural response

**Fig. 14:**
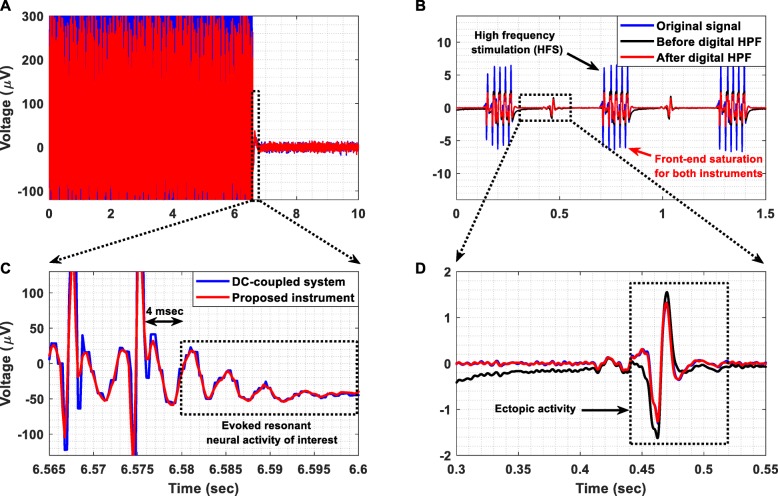
(**a**) Temporal response of the proposed instrument’s AFE when a signal segment containing ERNA, recorded by a dc-coupled commercial instrument from the STN at 2048 SPS, is injected to the instrument’s AFE. The signal was recorded by the instrument’s AFE and was then digitized by the Powerlab hardware at 4 kSPS (wired transmission method). (**b**) Temporal response recorded by the proposed instrument with and without the application of a real-time digital high-pass filter at 30 Hz when a signal that contains high-frequency stimulation (HFS) pulses and ectopic activity was injected to the biopotential recording AFE’s input by a waveform generator (Agilent 33220A). The signal was sampled by means of our instrument at 1 kSPS, was wirelessly transmitted to the receiver module and was depicted on the computer using the Powerlab 16/35 hardware (wireless transmission method). (**c**) Detailed view of the successfully recorded ERNA of interest. (**d**) Detailed view of the successfully recorded ectopic activity. It is clear that the application of a digital high-pass filter enables real-time recording of a high-quality signal, which approximates the signal recorded by the dc-coupled commercial medical device

In this experiment, a signal segment containing ERNA recorded by a dc-coupled commercial instrument from the STN at 2048 SPS, was injected at the instrument’s biopotential recording AFE by a commercial waveform generator (Agilent 33220A). The temporal response of the proposed instrument is shown in Fig. 14a. The main aim of this experiment was to ensure that: 1) no overshoot or ringing is produced by the ac-coupled AFE of the instrument as a response to DBS, and 2) the instrument’s AFE can reliably record the decaying oscillatory activity that characterizes the evoked potentials recorded from the STN. Indeed, our biopotential recording AFE exhibits a fast and free from any overshoot or ringing effects transient response to the stimulation pulses (see Fig. 14c). Hence, it can successfully record the ERNA of interest, which is considered to be the signal that appears 4 msec after the last DBS pulse and lasts for 20 msec in total (dotted rectangle in Fig. 14c) [20].

### Recording of PV ectopic activity

In this experimental procedure, a signal that contains HFS pulses and ectopic activity (represented by a solid blue line in Fig. 14b) was injected to the input of the proposed instrument by a waveform generator (Agilent 33220A). This signal was previously recorded (in bipolar mode from a catheter placed in the coronary sinus) using a commercially available wired and dc-coupled medical device with the sampling frequency set at 1 kSPS. Our instrument sampled the input signal at 1 kSPS and wirelessly transmitted it to the receiver module (wireless transmission method).

The temporal response of the proposed instrument with (solid red line) and without (solid black line) the application of a real-time digital high-pass filter at 30 Hz is presented in Fig. 14b. Clearly, the ac-coupled AFE of the proposed instrument exhibits a fast transient response (solid black line), which allows it to recover very quickly from the saturation state (+ 2.5 mV) it reached due to the high-amplitude HFS pulses and thus record the ectopic activity of interest. Moreover, as shown in Fig. 14d, a digital high-pass filter applied on the signal recorded by the proposed instrument rejected in real-time the dc offset induced by stimulation, producing an output (solid red line) that approximates the signal recorded by the commercial dc-coupled medical device. This measured result suggests that the application of a digital high-pass filter can significantly enhance the dc offset suppression capabilities of the AFE and increase the quality of the recorded signals. It is important to note here that the AFE of the commercial medical device also saturated during HFS because its maximum dynamic range was set by the clinicians at ±5 mV so that a sufficiently high gain is ensured.

### Recording of acceleration signals

**Fig. 15:**
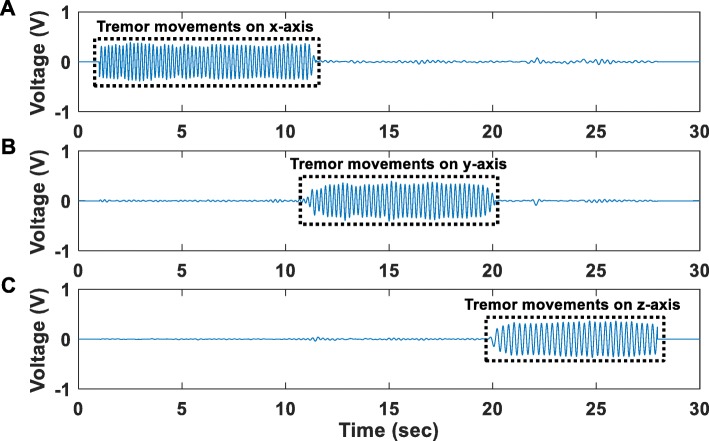
Time-domain profiles of acceleration signals recorded during three separate sessions. (**a**) during the first session, movements of the proposed instrument’s PCB, where the accelerometer is located, were produced along the x-axis for a duration of approximately 9 s (**b**) during the second session, movements of the proposed instrument’s PCB were produced along the y-axis for a duration of approximately 9 s (**c**) during the third session, movements of the proposed instrument’s PCB were produced along the z-axis for a duration of approximately 9 s. In all recording sessions, the instrument started from immobility and at the end of the produced movements it returned back to immobility. Clearly, under all examined circumstances (tremor movements in X, Y and Z axes), the accelerometer was able to successfully discriminate the state of immobility from the state where tremor occurs

In this experiment, acceleration signals recorded by the accelerometer located on the main PCB of the proposed instrument, were digitized at 1 kSPS and were wirelessly transmitted to the receiver module (wireless transmission method). Figure 15 shows the acceleration signals recorded during three separate sessions. During the first session, movements of the proposed instrument’s PCB, where the accelerometer is located, were produced on the x-axis for a duration of approximately 9 s (Fig. 15a). During the second session, movements of the proposed instrument’s PCB were produced on the y-axis for a duration of approximately 9 s (Fig. 15b). During the third session, movements of the proposed instrument’s PCB were produced on the z-axis again for a duration of approximately 9 s (Fig. 15c). In all recording sessions, the instrument started from immobility and at the end of the produced movements it returned back to immobility. Clearly, under all examined circumstances (tremor movements in X, Y and Z axes), the accelerometer was able to successfully discriminate the state of immobility from the state where tremor occurs.

### Comparison with other biosignal acquisition systems

The aim of the series of tests presented in this section was to compare the performance of the designed biopotential recording AFE with a commercial high-gain differential amplifier (model DP-301, Warner instruments) by injecting extremely weak biosignals to their inputs. More specifically, LFP signals were injected to the inputs of the two AFEs by a waveform generator (Agilent 33220A) and the quality of the output signals was assessed in both time and frequency domains. In the time domain, the normalized RMSE between the output of each AFE and the original LFP signal was used to evaluate the quality of biosignal recording. The normalization for the RMSE calculation was performed over the range of the reference signal, which is the original LFP signal.

**Fig. 16:**
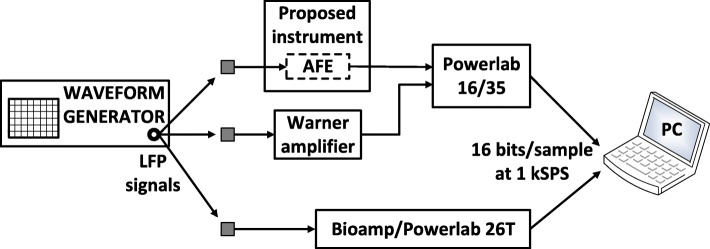
Recording of LFPs injected by a waveform generator (Agilent 33220A) to the inputs of: a) the proposed instrument’s AFE, b) a commercial high-gain differential amplifier (model DP-301, Warner instruments), and c) a very high-performance commercial bioamplifier (Powerlab 26 T, ADInstruments)

In order to compare the two AFEs on an equal footing, their analog outputs were digitized by the same ADC (ADC of the Powerlab 16/35 system, which provides a 16-bit resolution) at 1 kSPS. Next, a third device was introduced in the measurement setup (Bioamplifier/Powerlab 26 T) to record the same LFPs at 1 kSPS. The role of this device was to act as an independent reference instrument that is optimized for measuring weak bioelectrical signals such as EEG signals. The analog high-pass filters included in the DP-301 amplifier (cut-off frequency at 1 Hz) and the bioamplifier (cut-off frequency at 0.5 Hz) were activated so that their temporal responses can be compared with the temporal response of the proposed instrument’s ac-coupled AFE (cut-off frequency at 0.5 Hz) on an equal footing. Moreover, the gain of the DP-301 amplifier was set at 60 dB so that it is equal with the gain introduced by the biopotential recording AFE. Finally, the resolution of the reference instrument (bioamplifier/Powerlab 26 T) was set at ±100 μV (minimum available) during the LFP signal recording sessions. The measurement setup is shown in Fig. 16.

**Fig. 17:**
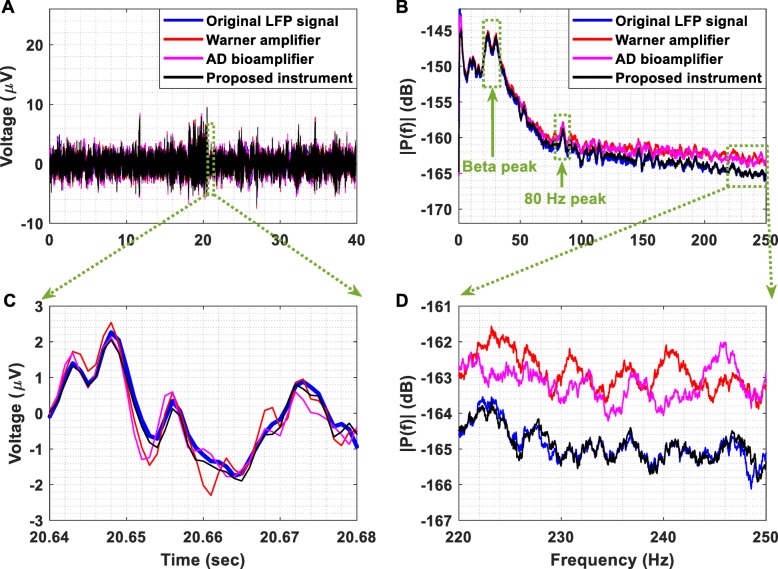
(**a**) Recording of LFPs. The original LFP signal, which was previously recorded from the STN in a patient with PD withdrawn from levodopa, was injected by a waveform generator (Agilent 33220A) to the inputs of the proposed instrument’s AFE, the DP-301 differential amplifier and the bioamplifier included in the Powerlab 26 T data acquisition system. (**b**) Amplitude spectrum of the signals presented in Fig. 17 (**a**). (**c**) The output of the proposed instrument’s AFE better tracks the changes occurring in the original LFP signal in comparison to the other two instruments. This is verified by the fact that the RMSE between the original LFP signal and the signal recorded by the proposed instrument’s AFE is less than the errors characterizing the other two instruments. (**d**) The detailed amplitude spectrum shows that the AFE of the proposed instrument provides accurate recording of the LFP signal, whereas the other two instruments cannot accurately record the frequencies of the LFP signal that are higher than 120 Hz

Referring to Fig. 17a and c, it is clear that the output of the proposed instrument’s AFE better tracks the changes occurring in the LFP signal presented at its input in comparison to the other two instruments. This is verified by the fact that the normalized RMSE between the original LFP signal and the signal recorded from the proposed instrument’s AFE is less than the errors characterizing the other two instruments. More specifically, the RMSE values that characterize the proposed instrument, the DP-301 amplifier and the bioamplifier are equal to 4, 4.1 and 4.9%.

According to the amplitude spectrum shown in Fig. 17b and d, the AFE of the proposed instrument provides accurate recording of the LFP signal, whereas the other two instruments cannot accurately record the frequencies of the LFP signal that are higher than 120 Hz. It is important to stress here that a significant portion of the calculated RMSE values can be attributed to the fact that four attenuators that provided 70 dB attenuation were used in order to bring the amplitude of the LFP signal injected by the waveform generator down to the level that characterizes the original LFP signal, which is approximately equal to 10 μV peak.

**Fig. 18:**
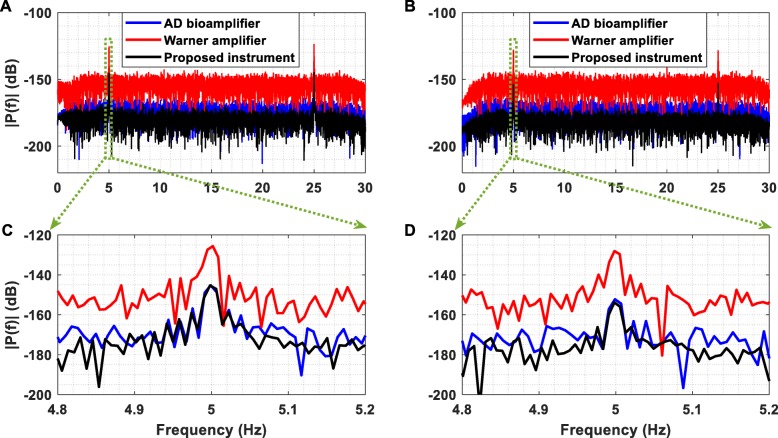
(**a**) Amplitude spectrum of the output voltage recorded from the AFE of the proposed instrument, the DP-301 Warner differential amplifier and the bioamplifier included in the Powerlab 26 T data acquisition system when two sinusoidal single tones (5 Hz and 25 Hz, amplitude 100 nV peak) were injected sequentially to the inputs of the instruments. The outputs of the three instruments were sampled at 1 kSPS. (**b**) Amplitude spectrum of the output voltage recorded from the thee instruments when two sinusoidal single tones (5 Hz and 25 Hz, amplitude 30 nV peak) were injected sequentially to the inputs of the instruments. The outputs of the three instruments were sampled at 1 kSPS. (**c**) It is clear that the AFE of the proposed instrument and the bioamplifier can accurately record the weak sinusoidal tone, whereas the DP-301 differential amplifier detects the tone but it cannot provide an accurate recording. (**d**) As in the case of the 100 nV peak sinusoidal tone, only the AFE of the proposed instrument and the bioamplifier can precisely record the 30 nV peak sinusoidal tone. It is important to note here that the noise floor of the proposed instrument is lower than the noise floors of the other two instruments

Furthermore, Fig. 18a exhibits the amplitude spectrum of the output voltage recorded from the AFE of the proposed instrument (black line), the DP-301 differential amplifier (red line) and the bioamplifier included in the Powerlab 26 T data acquisition system (pink line) when two sinusoidal single tones (5 Hz and 25 Hz, amplitude 100 nV peak) were injected sequentially to the inputs of the instruments and were sampled at 1 kSPS. To push the limits of the recording capabilities of the three instruments towards their noise floors, two weak sinusoidal single tones (5 Hz and 25 Hz, amplitude 30 nV peak) were injected sequentially to the inputs of the instruments and were also sampled at 1 kSPS (Fig. 18b).

Referring to Fig. 18c and d, it is clear that the AFE of the proposed instrument and the bioamplifier can accurately record both of the weak sinusoidal tones presented at their inputs, whereas the DP-301 differential amplifier detected the tones but it did not provide an accurate recording. Furthermore, Fig. 18 clearly shows that the noise floor of the proposed instrument is lower than the noise floors of the other two instruments. A comparison of the proposed instrument’s capabilities against the ones provided by the other two devices (DP-301 amplifier and Powerlab 26 T) is drawn in Table 3.

**Table 3:** Comparison of the proposed instrument with the Warner amplifier and the bioamplifier included in Powerlab 26 T

Parameters	Warner amplifier	Bioamp in Powerlab 26 T	This work
Number of channels	1	2	5 (+ 3)
Type of biosignals	EEG, ECG, extracellular spikes	ECG, EMG, EEG	ExG, ECoG, LFP, ERNA, body position and movement, PV ectopic activity (+ amperometric/potentiometric)
Supply of the ADC	–	±10 V	±2.5 V
Voltage gain	40–80 dB	54–100 dB	60 dB
Input voltage range	±1 mV to ±100 mV	±100 μV to ±20 mV	±2.3 mV
Maximum available bandwidth	DC to 10 kHz	Full bandwidth	0.5–500 Hz
Input impedance	1000 GΩ	100 MΩ	200 GΩ
Input referred noise	1.52 μV rms (1 Hz-10 kHz)	< 1 μV rms (0.5–2 kHz)	0.169 μV rms (0.5–500 Hz)
DC tolerance	±3 V	±300 mV	±85 mV
CMRR	100 dB	110 dB	134 dB
Maximum resolution of the ADC	Purely analog output	16 bits	24 bits
Maximum sampling frequency	Purely analog output	100 kSPS	1 kSPS (32 kSPS for wired)
Hours of continuous operation	200 (4 batteries 9 V)	Mains powered	8 (1 Ah lithium battery)
Wireless capability	No	No	Yes (Zigbee)
Area (length × width)	350 cm^2^	500 cm^2^	44 cm^2^ (PCB area)

Another important factor that needed to be examined was the latency added by the wireless transmission method for the wireless transmission of eight channels of data. This latency was measured and found to be approximately equal to 1 msec. It is attributed to the time required for the wireless transmission (= 512 μsec) and the time needed for the serial peripheral interface (SPI) communication between the other blocks of the instrument’s design. More specifically, 36 μsec are needed for the ADS1298-FPGA communication, 107 μsec for the FPGA-Tx communication, 36 μsec for the Rx-FPGA communication and finally another 32 μsec for the FPGA-DAC communication (in total 723 μsec). Moreover, we have confirmed the integrity and reliability of the wireless biosignal recordings for a distance between the proposed instrument and the receiver module up to 5 m. Finally, it should be noted here that no difficulties were observed in normal operation of our system when the wearable/wireless device and the receiver module were placed in two different adjacent rooms that were separated by a wall.

A comparison of the proposed instrument with (portable and implantable) wireless commercial biopotential acquisition systems is given in Table 4. This work demonstrates the highest CMRR and input impedance, the lowest input referred noise and smallest size among the state-of-the-art instruments presented in this table. Moreover, compared to the TMSI Mobita device, it can provide 8 h of continuous operation (continuous wireless transmission of all eight channels of the simultaneous-sampling ADC included in our instrument) with a smaller lithium battery. This is attributed to the fact that Mobita uses the Wi-Fi protocol for wireless data transmission, which allows for a higher maximum sampling rate (2000 SPS) at the expense of higher power consumption and shorter battery life. It is important to highlight here that, to the best of our knowledge, there are no commercial, small, battery-powered, wearable and wireless recording-only instruments that claim the capability of recording ECoG signals. As shown in Table 4, it is clear that, compared to high-performance bidirectional interface systems (such as the Activa PC + S neurostimulator from Medtronic), which are widely used in applications that include concurrent sensing and closed-loop neurostimulation, our instrument is better suited for applications where multi-channel (> 4 channels) and high-resolution (> 10 bits) ECoG recording (without stimulation) is required.

**Table 4:** Comparison of the proposed instrument with commercial (portable and implantable) wireless biopotential acquisition systems

Parameters	TMSI Mobita	TMSI Mobi	Activa PC + S	This work
Number of channels	32	6 (+ 4)	2	5 (+ 3)
Type of biosignals	ExG, body position and movement	ExG, (+ temperature, respiration, body position and movement)	LFP/ECoG	ExG, ECoG, LFP, ERNA, body position and movement, PV ectopic activity (+ amperometric/potentiometric)
Supply of the ADC	±2 V	±2 V	1.7–2.2 V	±2.5 V
Voltage gain	20 dB	26 dB	48–66 dB	60 dB
Input voltage range	±200 mV	±100 mV	±500 μV to ±4.4 mV (for 2.2 V supply)	±2.3 mV
Maximum available bandwidth	DC up to 0.13 × sample frequency	–	0.5–260 Hz	0.5–500 Hz
Input impedance	> 100 MΩ	> 100 MΩ	–	200 GΩ
Input referred noise	< 0.4 μV rms (0.1–10 Hz)	< 1 μV rms	< 1 μV rms	0.169 μV rms (0.5–500 Hz)
DC tolerance	–	–	–	±85 mV
CMRR	> 100 dB	> 90 dB	> 80 dB	134 dB
Maximum resolution of the ADC	24 bits	24 bits	10 bits	24 bits
Maximum sampling frequency	2000 SPS	2048 SPS	422 SPS	1 kSPS (32 kSPS for wired)
Hours of continuous operation	6 to 8 (4.1 Ah lithium battery)	- (2 AA batteries)	–	8 (1 Ah lithium battery)
Wireless capability	Yes (Wi-Fi)	Yes (Bluetooth)	Yes (175 kHz near-field inductive)	Yes (Zigbee)
Area (length × width)	105 cm^2^	112 cm^2^	39 cm^2^	44 cm^2^ (PCB area)

Moreover, a comparison of the proposed instrument with other state-of-the-art wearable and wireless biopotential acquisition systems that exist in the literature and also use discrete (commercial) components as building blocks is performed in Table 5. These systems can record biopotential signals of a specific type by using either commercial analog front-end chips (e.g. the Intan RHD2132 chip in [43] or the ADS1299 chip in [44]) or commercially available components to build application-specific analog front-ends (e.g. in this work and in [45]). It is clear that our instrument achieves a noise performance that is significantly better than the noise performance provided by the other three devices. It is important to emphasize here that although two of the devices presented in Table 5 (in [44, 45]) offer lower bandwidth than our device, they still provide an integrated noise value that is higher than the one offered by our instrument. The versatility of our AFE architecture and the real-time wireless biosignal transmission it can offer at 1 kSPS sampling frequency allows the proposed instrument to record a wider variety of biosignals compared to the other systems, with the lowest wireless transmission latency. In addition, it offers the highest input impedance, which allows it to efficiently interface with high-impedance electrodes (e.g. segmented electrodes in DBS), and the highest CMRR value, which can be very useful in applications where large common-mode disturbances (stemming from the application of strong stimulation pulses in simultaneous biosignal recording and stimulation setups) have to be rejected. The remaining features of our device are equally good or comparable to the features of the other wearable and wireless systems presented in Table 5.

**Table 5:** Comparison of the proposed instrument with other state-of-the-art wearable and wireless biopotential acquisition systems existing in the literature

Parameters	[43]	[44]	[45]	This work
Number of channels	32	8	4	5 (+ 3)
Type of biosignals	EMG	ECG, EMG	EEG, LFP	ExG, ECoG, LFP, ERNA, body position and movement, PV ectopic activity (+ amperometric/potentiometric)
Voltage gain	46 dB	≈ 28 dB (max)	≈ 54 dB	60 dB
Input voltage range	±5 mV	±100 mV	±1.15 mV	±2.3 mV
Maximum available bandwidth	10–500 Hz	0–250 Hz	1.5–100 Hz	0.5–500 Hz
Input impedance	1.3 GΩ (10 Hz)	≈ 620 MΩ (10 Hz)	47 kΩ (10 Hz)	≈ 8 GΩ (10 Hz)
Input referred noise	< 3 μV rms	0.2 μV rms (0–250 Hz)	≈ 0.606 μV rms (1.5–100 Hz)	0.169 μV rms (0.5–500 Hz)
CMRR	82 dB	110 dB	–	134 dB
Maximum resolution of the ADC	16 bits	24 bits	16 bits	24 bits
Maximum sampling frequency	2048 SPS	500 SPS (16 kSPS for wired)	500 SPS	1 kSPS (32 kSPS for wired)
Hours of continuous operation	5 (600 mAh 1-Cell LiPo battery)	13.6 (1700 mAh battery)	6 to 8 (CR1/3 N lithium ion button-cell battery)	8 (1 Ah lithium battery)
Wireless capability	Yes (Wi-Fi)	Yes (Bluetooth)	Yes (2.4 GHz MSK)	Yes (Zigbee)
Latency	12 ms	–	–	1 ms
Max. transmission range	22 m	10 m	3–5 m	5 m
Area (length × width)	10.2 cm^2^	21.7 cm^2^ (PCB area)	4.76 cm^2^	44 cm^2^ (PCB area)

Finally, a comparison of our AFE with state-of-the-art ASICs for biopotential signal acquisition is given in Table 6. As anticipated, a tradeoff exists between ultra-low power consumption (state-of-the-art ASICs) and superior noise performance (proposed instrument’s AFE).

**Table 6:** Comparison of the proposed instrument’s AFE with state-of-the-art ASICs for biopotential signal acquisition (note that, as anticipated, a tradeoff exists between ultra-low power consumption (state-of-the-art ASICs) and superior noise performance (proposed instrument’s AFE))

Parameters	[46]	[47]	[48]	[49]	[50]	This work
Supply	0.2/0.8 V	1 V	1.8 V	1.8 V	3 V	±5 V
Technology	0.18 μm CMOS	0.18 μm CMOS	0.8 μm CMOS	0.18 μm CMOS	0.5 μm CMOS	–
Voltage gain	776 V/V	100 V/V	100 V/V	3–100 V/V	10 V/V	1000 V/V
Input impedance	≈ 100 MΩ	> 700 MΩ	> 7.5 MΩ	> 2 GΩ	> 100 MΩ	200 GΩ
Input referred noise	0.94 μV rms (0.5–670 Hz)	1.3 μV rms (0.5–100 Hz)	0.95 μV rms (0.05–100 Hz)	0.8 μV rms (0.5–100 Hz)	0.6 μV rms (0.5–100 Hz)	85 nV rms (0.5–100 Hz)
DC tolerance	–	Rail-to-rail	±50 mV	Rail-to-rail	±50 mV	±85 mV
CMRR	85 dB	60 dB	100 dB	82 dB	120 dB	134 dB
Current	≈ 1 μA	3.5 μA	1.1 μA	11 μA	11.1 μA	≈ 2.5 mA

## Discussion

The system architecture validated in this study addresses several of the major challenges to the development of a small, high-precision, battery-powered, wireless and wearable multi-instrument that is intended to be used in the ICU or in a High Dependency Unit, or in patient home monitoring studies. A difficult task when designing a biopotential acquisition AFE that is intended to be used in a clinical setting, is to ensure that it can provide real-time biosignal recordings. This real-time character is particularly important for neuromodulation because the stimulation must change in real time based on the measured state of the neural network [16].

The approach adopted in this work was to use a high-gain (=100 V/V) front-end INA in order to maintain high CMRR values (=134 dB), and add an active feedback integrator to provide high-pass characteristics at the first stage of the AFE. Next, an active 1st order high-pass filter and a passive 2nd order low-pass filter were introduced in the following stages of the AFE in order to ensure that an adequate suppression of dc electrode offsets and high-frequency noise components is achieved in real time. The merits of this approach are: 1) significant delays in data processing, which are introduced in the signal chain by the application of (usually high-order) digital filtering techniques, are avoided, 2) front-end amplifier saturation issues are prevented. These issues often emerge in architectures where a high front-end gain is applied (to make the weak biosignals detectable by the front-end electronics and the subsequent ADC blocks) and analog filtering blocks are absent from the system design since the signal conditioning process only takes place in the digital domain, and 3) the introduction of front-end passive filters, which can lead to the degradation of the combined (passive filter plus differential amplifier) apparent CMRR of the front-end due to component mismatches [51], is avoided.

Clearly, by assigning the task of electrode dc offset and high-frequency noise suppression to the analog domain, we ensure that the real-time character of the system is maintained. Measured experimental results show that this aim has been achieved without significantly increasing the power consumption (at least 8 h of continuous operation is provided) and size (equal to the size of a business card) of the designed instrument. The proposed AFE consists of three active components per channel, namely one INA and two operational amplifiers, and its current consumption is approximately equal to 2.5 mA. Moreover, all of the imposed requirements on the AFE design, which were presented in Table 2, have been satisfied. Further improvements in the dc electrode offset rejection capabilities of the instrument that will enhance its performance in applications where strong stimulation artefacts affect the quality of the recorded signals (e.g. DBS, HFS, etc.) can be accomplished in the digital domain by applying real-time high-pass filters (see Fig. 14d).

By adding a DAC on the receiver module, we ensure that the high-quality signals recorded and wirelessly transmitted by the proposed instrument can be successfully recorded and depicted on the computer by commercially available data acquisition systems that are widely used in clinical settings. This feature renders the proposed instrument an assistive device that can functionally integrate with existing biosignal recording systems to offer wireless communication, while maintaining signal integrity. Indeed, in this study, the biosignals recorded and wirelessly transmitted by the proposed instrument were received by the receiver module and were successfully depicted on the computer using the graphical user interface of a commercially available data acquisition system (Powerlab 16/35, ADInstruments) widely used by clinicians.

However, having ensured that the proposed instrument can offer high-quality and real-time recordings of a wide variety of bioelectrical signals, the next step towards rendering it a functional instrument that can further facilitate the process of clinical decision-making, would be to add a wired communication capability in order to cover the extra need that may exist for high sampling frequency (the maximum sampling frequency offered by the ADS1298 chip is 32 kSPS) at the expense of restricted mobility. In this case, the data sampled by the ADS1298 chip could be directed from the FPGA to the computer using the USB 2.0 interface that is provided by the FPGA module. Finally, the current system design utilizes only a small fraction of the available FPGA resources. It is thus clear that there is still ample room for implementing closed-loop algorithms and digital FIR filtering blocks to further improve computational efficiency.

## Conclusions

The novel, versatile and state-of-the-art instrument designed, realized and tested both ex vivo and in vivo allows for low-noise, real-time and wireless recording of a plethora of bioelectrical signals for a bandwidth of 0.5–500 Hz. Proof of the proposed instrument’s recording capabilities has been provided and its performance has been evaluated quantitatively by means of a series of tests and comparisons with other high-performance biopotential acquisition systems (both commercial devices and academic works were used for this purpose).

Since the proposed instrument is small in size (≈ area of a business card), battery-powered, wearable and wireless, it could be used to alleviate the problem of limited mobility encountered by patients due to the fact that they are connected to bulky and mains-powered instruments. Hence, it could allow for continuous monitoring of patients, thus increasing the biosignal acquisition time and enhancing the diagnostics of various diseases (such as PD, essential tremor, epilepsy, ALS, AF, traumatic brain injury, cardiovascular disease, etc.). Furthermore, since the spectral content of some types of biosignals (e.g. LFPs) varies among patients [18], the extended passband offered by the proposed device could lead to a more in-depth analysis of the spectral content recorded from different patients, facilitating the personalization of treatment for patients suffering from serious diseases, such as PD.

Moreover, the enhanced recording capabilities of this instrument, stemming from its low input referred noise (8 nV/√Hz), have the potential to reveal biomarkers of various neurological disorders existing in weak neural oscillations, previously hidden by the inherent noise of older biopotential acquisition systems. Hence, this tool may allow for a deeper understanding of disease mechanisms, physiology and neural processing. Finally, the proposed instrument can be used for the determination of features extracted from ECoG/LFP signals that could serve as biomarkers for regulating and optimizing ongoing DBS. Hence, among others, this work paves the way for the development of a portable/wearable closed-loop neurostimulation modality that uses low and higher-frequency ECoG/LFPs as control signals.

## Data Availability

Data sharing is not applicable to this article as no datasets were generated or analysed during the current study.

## Abbreviations

ADC: Analog-to-digital converter
AF: Atrial fibrillation
AFE: Analog front-end
ALS: Amyotrophic lateral sclerosis
ASICs: Application-specific integrated circuits
BER: Bit error rate
CMRR: Common mode rejection ratio
DAC: Digital-to-analog converter
DBS: Deep brain stimulation
ECG: Electrocardiographic
ECoG: Electrocorticographic
EEG: Electroencephalographic
EMG: Electromyographic
EMI: Electromagnetic interference
ERNA: Evoked resonant neural activity
FIR: Finite impulse response
FPGA: Field-programmable gate array
GP: Ganglionated plexi
HFS: High frequency stimulation
ICU: Intensive care unit
IMD: Intermodulation distortion
IMD_3_: Third-order intermodulation distortion
INA: Instrumentation amplifier
IP_3_: Third-order intercept point
LFPs: Local field potentials
MSK: Minimum Shift Keying (modulation scheme)
PC: Personal computer
PCB: Printed circuit board
PD: Parkinson’s disease
PV: Pulmonary vein
RMSE: Root mean square error
SMA: Sub-miniature version A
SNR: Signal-to-noise ratio
SPI: Serial peripheral interface
STN: Subthalamic nucleus
THD: Total harmonic distortion
USB: Universal serial bus

## References

[1] Abdelhalim K, Jafari HM, Kokarovtseva L, Velazquez JLP, Genov R (2013). 64-channel uwb wireless neural vector analyzer soc with a closed-loop phase synchrony-triggered neurostimulator. IEEE J Solid State Circuits.

[2] Schnitzler A, Gross J (2005). Normal and pathological oscillatory communication in the brain. Nat Rev Neurosci.

[3] Uhlhaas PJ, Singer W (2006). Neural synchrony in brain disorders: relevance for cognitive dysfunctions and pathophysiology. Neuron.

[4] Autism and Developmental Disabilities Monitoring Network Surveillance Year (2008). Principal investigators, Centers for Disease Control and Prevention (2012) prevalence of autism spectrum disorders--autism and developmental disabilities monitoring network, 14 sites, United States, 2008. MMWR Surveill Summ.

[5] Wittchen HU, Jacobi F, Rehm J (2011). The size and burden of mental disorders and other disorders of the brain in Europe 2010. Eur Neuropsychopharmacol.

[6] Holleman Jeremy, Zhang Fan, Otis Brian (2011). Ultra Low-Power Integrated Circuit Design for Wireless Neural Interfaces.

[7] Denison Tim, Consoer Kelly, Santa Wesley, Avestruz Al-Thaddeus, Cooley John, Kelly Andy (2007). A 2 $\mu\hbox{W}$ 100 nV/rtHz Chopper-Stabilized Instrumentation Amplifier for Chronic Measurement of Neural Field Potentials. IEEE Journal of Solid-State Circuits.

[8] Wessberg J, Stambaugh CR, Kralik JD, Beck PD, Laubach M, Chapin JK, Kim J, Biggs SJ, Srinivasan MA, Nicolelis MAL (2000). Real-time prediction of hand trajectory by ensembles of cortical neurons in primates. Nature.

[9] Harrison RR, Charles C (2003). A low-power low-noise CMOS amplifier for neural recording applications. IEEE J Solid State Circuits.

[10] Schwartz AB, Cui XT, Weber DJJ, Moran DW (2006). Brain-controlled interfaces: movement restoration with neural prosthetics. Neuron.

[11] Heldman DA, Wang W, Chan SS, Moran DW (2006). Local field potential spectral tuning in motor cortex during reaching. IEEE trans.

[12] Pagkalos I, Rogers ML, Boutelle MG, Drakakis EM (2018). A high-performance application specific integrated circuit for electrical and neurochemical traumatic brain injury monitoring. ChemPhysChem.

[13] Papadimitriou KI, Wang C, Rogers ML, Gowers SAN, Leong CL, Boutelle MG, Drakakis EM (2016). High-performance bioinstrumentation for real-time Neuroelectrochemical traumatic brain injury monitoring. Front Hum Neurosci.

[14] Litt B, D’Alessandro A, Esteller R, Echauz J, Vachtsevanos G (2003) Translating seizure detection, prediction and brain stimulation into implantable devices for epilepsy. In: Int. IEEE/EMBS Conf. Neural Eng. NER. IEEE, pp 485–488.

[15] Leuthardt EC, Schalk G, Wolpaw JR, Ojemann JG, Moran DW (2004). A brain-computer interface using electrocorticographic signals in humans. J Neural Eng.

[16] Stanslaski S, Afshar P, Cong P, Giftakis J, Stypulkowski P, Carlson D, Linde D, Ullestad D, Avestruz AT, Denison T (2012). Design and validation of a fully implantable, chronic, closed-loop neuromodulation device with concurrent sensing and stimulation. IEEE Trans Neural Syst Rehabil Eng.

[17] Little S, Pogosyan A, Neal S (2013). Adaptive deep brain stimulation in advanced Parkinson disease. Ann Neurol.

[18] Arlotti M, Rossi L, Rosa M, Marceglia S, Priori A (2016). An external portable device for adaptive deep brain stimulation (aDBS) clinical research in advanced Parkinson’s disease. Med Eng Phys.

[19] Priori A, Foffani G, Rossi L, Marceglia S (2013). Adaptive deep brain stimulation (aDBS) controlled by local field potential oscillations. Exp Neurol.

[20] Sinclair NC, McDermott HJ, Bulluss KJ, Fallon JB, Perera T, Xu SS, Brown P, Thevathasan W (2018). Subthalamic nucleus deep brain stimulation evokes resonant neural activity. Ann Neurol.

[21] Mills KR (2010). Characteristics of fasciculations in amyotrophic lateral sclerosis and the benign fasciculation syndrome. Brain.

[22] Hebb AO, Zhang JJ, Mahoor MH, Tsiokos C, Matlack C, Chizeck HJ, Pouratian N (2014). Creating the feedback loop. Closed-loop Neurostimulation. Neurosurg Clin N Am.

[23] Eyuboglu M, Karabag Y, Karakoyun S, Senarslan O, Tanriverdi Z, Akdeniz B (2017). Usefulness of fragmented QRS in hypertensive patients in the absence of left ventricular hypertrophy. J Clin Hypertens.

[24] Nguyen KT, Vittinghoff E, Dewland TA, et al. Ectopy on a single 12-Lead ECG, incident cardiac myopathy, and death in the community. J Am Heart Assoc. 2017. 10.1161/JAHA.117.006028. 10.1161/JAHA.117.006028 PMC5586444 28775064

[25] Lim PB, Malcolme-Lawes LC, Stuber T, Wright I, Francis DP, Davies DW, Peters NS, Kanagaratnam P (2011). Intrinsic cardiac autonomic stimulation induces pulmonary vein ectopy and triggers atrial fibrillation in humans. J Cardiovasc Electrophysiol.

[26] Schauerte P, Scherlag BJ, Pitha J, Scherlag MA, Reynolds D, Lazzara R, Jackman WM (2000). Catheter ablation of cardiac autonomic nerves for prevention of vagal atrial fibrillation. Circulation.

[27] Schauerte P, Scherlag BJ, Patterson E, Scherlag MA, Matsudaria K, Nakagawa H, Lazzara R, Jackman WM (2001). Focal atrial fibrillation: experimental evidence for a pathophysiologic role of the autonomic nervous system. J Cardiovasc Electrophysiol.

[28] Lin J, Scherlag BJ, Zhou J, Lu Z, Patterson E, Jackman WM, Lazzara R, Po SS (2007). Autonomic mechanism to explain complex fractionated atrial electrograms (CFAE). J Cardiovasc Electrophysiol.

[29] Zhou J, Scherlag BJ, Edwards J, Jackman WM, Lazzara R, Po SS (2007). Gradients of atrial refractoriness and inducibility of atrial fibrillation due to stimulation of ganglionated plexi. J Cardiovasc Electrophysiol.

[30] Yazıcıoğlu Refet Fırat, Van Hoof Chris, Puers Robert (2009). Biopotential Readout Circuits for Portable Acquisition Systems.

[31] Rossi PJ, Gunduz A, Judy J (2016). Proceedings of the third annual deep brain stimulation think tank: a review of emerging issues and technologies. Front Neurosci.

[32] Buhlmann J, Hofmann L, Tass PA, Hauptmann C. Modeling of a segmented electrode for desynchronizing deep brain stimulation. Front Neuroeng. 2011. 10.3389/fneng.2011.00015. 10.3389/fneng.2011.00015 PMC3233722 22163220

[33] Wei XF, Grill WM (2005). Current density distributions, field distributions and impedance analysis of segmented deep brain stimulation electrodes. J Neural Eng.

[34] Merrill DR, Bikson M, Jefferys JGR (2005). Electrical stimulation of excitable tissue: design of efficacious and safe protocols. J Neurosci Methods.

[35] Nikola J, Ratko P, Strahinja D, Dejan PB (2001) A novel AC-amplifier for electrophysiology: active DC suppression with differential to differential amplifier in the feedback-loop. In: Annu. Reports Res. React. Institute, Kyoto Univ. IEEE, pp 3328–3331.

[36] Northrop RB. Analysis and application of analog electronic circuits to biomedical instrumentation: CRC Press; 2012.

[37] Poshala P, KK R, Gupta R (2014) Signal chain noise figure analysis. Texas Instruments SLAA652:1–14.

[38] Cagnan H, Pedrosa D, Little S (2017). Stimulating at the right time: phase-specific deep brain stimulation. Brain.

[39] Lee J-S, Su Y-W, Shen C-C (2007). A comparative study of wireless protocols: Bluetooth, UWB, ZigBee, and Wi-fi. IECON 2007 - 33rd Annu.

[40] Petrova M, Riihijarvi J, Mahonen P, Labella S (2006) Performance study of IEEE 802.15.4 using measurements and simulations. In: IEEE Wirel. Commun. Netw. Conf. 2006. WCNC 2006. IEEE, pp 487–492.

[41] Zumbahlen Hank (2008). Analog Filters. Linear Circuit Design Handbook.

[42] Francis J (2016). ECG monitoring leads and special leads. Indian Pacing Electrophysiol J.

[43] Cerone Giacinto Luigi, Botter Alberto, Gazzoni Marco (2019). A Modular, Smart, and Wearable System for High Density sEMG Detection. IEEE Transactions on Biomedical Engineering.

[44] Sarker VK, Jiang M, Gia TN, Anzanpour A, Rahmani AM, Liljeberg P (2017) Portable multipurpose bio-signal acquisition and wireless streaming device for wearables. In: SAS 2017–2017 IEEE Sensors Appl. Symp. Proc. IEEE, pp 1–6.

[45] Pinnell RC, Dempster J, Pratt J (2015). Miniature wireless recording and stimulation system for rodent behavioural testing. J Neural Eng.

[46] Yaul FM, Chandrakasan AP (2017). A noise-efficient 36 nV/√Hz chopper amplifier using an inverter-based 0.2-V supply input stage. IEEE J Solid State Circuits.

[47] Verma N, Shoeb A, Bohorquez J, Dawson J, Guttag J, Chandrakasan AP (2010). A micro-power EEG acquisition SoC with integrated feature extraction processor for a chronic seizure detection system. IEEE J Solid State Circuits.

[48] Denison T, Consoer K, Kelly A, Hachenburg A, Santa W (2007) A 2.2μW 94nV/√Hz, chopper-stabilized instrumentation amplifier for EEG detection in chronic implants. In: dig. Tech. Pap. - IEEE Int. solid-state circuits Conf. IEEE, pp 162–594.

[49] Xu J, Yazicioglu RF, Grundlehner B, Harpe P, Makinwa KAA, Van Hoof C (2011). A 160 μw 8-channel active electrode system for EEG monitoring. IEEE trans.

[50] Yazicioglu RF, Merken P, Puers R, Van Hoof C (2007). A 60 μW 60 nV/√Hz readout front-end for portable biopotential acquisition systems.

[51] Casas O, Spinelli EM, Pallàs-Areny R (2009). Fully differential AC-coupling networks: a comparative study. IEEE trans.

